# Anti-Obesity Effect of the Above-Ground Part of *Valeriana dageletiana* Nakai ex F. Maek Extract in High-Fat Diet-Induced Obese C57BL/6N Mice

**DOI:** 10.3390/nu9070689

**Published:** 2017-07-02

**Authors:** Zhiqiang Wang, Seung Hwan Hwang, Ju Hee Kim, Soon Sung Lim

**Affiliations:** 1 Department of Preventive Medicine and Health Management, Hebei University, Baoding 071002, China; wangzq01234@gmail.com; 2 Department of Food Science and Nutrition, Hallym University, Chuncheon 24252, Korea; isohsh@gmail.com; 3 Institute of Natural Medicine, Hallym University, Chuncheon 24252, Korea; 3206kjhhjk@hanmail.net; 4 Institute of Korean Nutrition, Hallym University, Chuncheon 24252, Korea

**Keywords:** 3T3-L1, herbal medicine, epididymal adipose tissue, fatty liver, adipogenesis, lipogenesis, lipid profile

## Abstract

*Valeriana dageletiana* Nakai ex F. Maek (VD) has been used as traditional medicine for the treatment of restlessness and sleeping disorders. However, it is still unclear whether obesity in mice can be altered by diet supplementation with VD. In this study, we first investigated the influences of VD on the accumulation of lipid content in 3T3-L1 cells; and the results showed that the above-ground VD extracts (VDAE) suppressed the differentiation of 3T3-L1 preadipocytes in a concentration-dependent manner without cytotoxicity. Thus, the effects of VDAE on preventing obesity were then studied in the C57BL/6N mice for 10 weeks (*n* = 6): normal-fat diet, high-fat diet (HFD), HFD supplemented with 1% (10 g/kg) *Garcinia combogia* extract (positive control), and HFD supplemented with 1% (10 g/kg) VDAE. The results showed that VDAE reduced food efficiency ratio, body weight, epididymal adipose and hepatic tissue weight, hepatic lipid metabolites, and triacylglycerol and cholesterol serum levels compared to the high-fat diet group. Moreover, VD significantly inhibited the expression of adipogenic genes, such as *PPAR-γ*, *C/EBP-α*, and *aP2*, and lipogenic genes, such as *SREBP-1c*, *FAS*, *SCD-1*, and *CD36*, in epididymal adipose tissue and hepatic tissue. These findings indicate anti-adipogenic and anti-lipogenic effects of VDAE and suggest that it could be a potent functional food ingredient for the prevention of high-fat diet-induced obesity.

## 1. Introduction

Since 1980, the prevalence of obesity has increased and accelerated worldwide [1] and more than one billion people are expected to be obese by the year 2020 [2]. Obesity results from an energy balance disorder between calorie intake and energy excess, in which extra energy is stored as triglyceride through adipogenesis and lipid accumulation in adipose tissue, liver, and other organs [3]. Obesity induced by adipogenesis and lipid accumulation in organelles can cause diabetes in 44% of cases, ischemic heart disease in 23% of cases, and specific cancers in 7–41% of cases [4]. Adipogenesis is a process of cell differentiation by which undifferentiated pre-adipocytes become mature adipocytes. Many hormones, nutrients, and transcription factors regulate lipid accumulation during adipocyte differentiation [5]. Furthermore, the regulation of adipogenic transcriptional factor mRNA levels, such as *peroxisome proliferator-activator receptor-γ* (*PPAR-γ*), *CCAAT/enhancer binding protein-α* (*C/EBP-α*), *sterol regulatory element-binding protein* (*SREBP-1c*), and related genes (*aP2*, *FAS*), plays a key role in the process of adipogenesis [6,7,8]. Lipogenesis is a process that encompasses fatty acid and triglyceride synthesis in adipose tissue and in the liver. The fat synthesis and storage in the liver are generally induced by high-fat diets, high cholesterol diets, fructose-enriched diets, alcoholic beverages, etc. [9,10,11,12]. Moreover, regulation of the production of lipogenic enzymes, such as lipoprotein lipase (LPL), fatty acid synthase (FAS), and acetyl-CoA carboxylase (ACC) and fatty acid esterification transcriptional factors, such as stearoyl-CoA desaturase (SCD-1), is important in the process of lipogenesis in adipose and hepatic tissues [13].

Valerian, which is a perennial plant of Valerianaceus family, widely grows wild in Europe, North and South America, and parts of Northern Asia. It favors growing in humid woods and by streams and rivers. As bushes, the height of valerians range from 1–1.5 m [14]. Camphene, α-therpineol, azulene, geraniol, borneol, β-caryophyllene, some ketones, some esters (i.e*.*, bornyl acetate, bornyl-isovalerate, bornyl-formiate, eugenyl-isovalerate, and isoeugenyle), and valeric and isovaleric acids are the major components of valerian species reported in previous literature. Additionally, non-glycosidic iridoid esters (normally known as valepotriates), of which cytotoxic and mutagenic effects have been reported, mainly in valtrate and hydro-valtrate, are another important group of active compounds existing in valerians. Valerians also contain alkaloids, like chatinine, valerine, valerianine, isovaleriamide, and actinidine [14]. *Valeriana dageletiana* Nakai ex F. Maek. (VD), wide-leaf valerian, is a perennial herb that grows on Ulleung Island in Korea. The root of the plant has been used in sedative infusions and for nervous system sicknesses in allopathic and homeopathic medicine, while the upper part of the plant (stem and leaf) has been used as food. Valerian species have been reported to have antibacterial and anti-oxidant activities [15], and can also be used in the treatment of restlessness and sleeping disorders [16]. However, to our knowledge, studies have not yet reported the anti-obesity effects of VD extracts.

To determine whether obesity in mice can be ameliorated by diet supplementation with VD, in this study, the anti-adipogenic effects of extracts from the upper part of the plant (stem and leaf) and root of VD were first investigated and compared in 3T3-L1 adipocytes; furthermore, we examined the anti-obesity effects of the extract from the upper part of VD, known as the edible part of the plant, in high-fat-diet-induced obese mice.

## 2. Materials and Methods

### 2.1. Plant Material and Preparation of the Extract

The whole plant of VD was harvested from Ulleung Island in May 2015. The dried above-ground (stem and leaf) and below-ground (root) parts of VD (1.5 kg) were each pulverized and then were extracted using 70% ethanol (15 L) at room temperature for 48 h. The VD extracts from above-ground (VDAE) and below-ground (VDBE) were filtered using filter paper (Hyundai Micro No. 20, Bucheon, Korea) and concentrated by a reduced pressure evaporator (N-1000, Tokyo Rikakikai, Tokyo, Japan), and then finally freeze-dried using PVTFD10R (Ilshinbiobase Co., Ltd., Yangju, Korea) to obtain extract powder.

### 2.2. 3T3-L1 Cell Culture and Treatment

3T3-L1 murine pre-adipocytes were obtained from American Type Culture Collection (Manassas, VA, USA) and cultured to confluency at 37 °C under a humidified 5% CO_2_ atmosphere in Dulbecco’s Modified Eagle’s Medium (DMEM, Gibco, Waltham, MA, USA), including 10% bovine calf serum (GenDEPOT, Katy, TX, USA) and 100 U/mL penicillin-streptomycin (Gibco). Two days after the cells had reached confluency (day 0), pre-adipocytes of 3T3-L1 were cultured in differentiation medium (DM) containing 10% fetal bovine serum (FBS, Gibco), 10 μg/mL insulin (Sigma-Aldrich, St. Louis, MO, USA), 0.5 mM 3-isobutyl-1-methyxanthine (IBMX, Sigma-Aldrich), and 1 μM dexamethasone (Sigma-Aldrich). Two days after stimulation with a differentiation inducer (MDI, including 0.5 mM IBMX, 1 μM dexamethasone and 10 μg/mL insulin) (day 2), the medium was converted to 10% FBS/DMEM medium containing 10 μg/mL insulin. After two days (day 4), the medium was changed to 10% FBS/DMEM medium and cultured in 10% FBS/DMEM medium every two days. Full differentiation was achieved by day 8. During differentiation, the VD extracts were treated to inhibit the differentiation of adipocytes on 3T3-L1 culture at concentrations of 10 and 50 μg/mL between days 0 and 4.

### 2.3. Oil Red O Staining and Determination of Lipid Content

To investigate both adipogenic potential and lipid accumulation, cells were stained with Oil Red O solution (Sigma-Aldrich). On day 8, the cultured 3T3-L1 cells were washed with cold phosphate-buffered saline (PBS) and then fixed with 10% formaldehyde at room temperature. The cells were stained with filtered 0.5 μg/mL Oil Red O solution (0.5 g of Oil Red O in 500 mL of isopropyl alcohol) and washed twice. The lipid droplets were dissolved in isopropanol and absorbance was measured at 540 nm using a microplate reader (Sensident Scan, Labsystems, Helsinki, Finland).

### 2.4. Cell Viability Assay

The cell viability of VD extracts in 3T3-L1 cells was investigated using an 3-(4,5-dimethylthiazol-2-yl)-5-(3-carboxymethoxyphenyl)-2-(4-sulfophenyl)-2*H*-tetrazolium, inner salt (MTS) assay kit (Promega, Madison, WI, USA) according to the manufacturer’s instructions. 3T3-L1 cells (5 × 10^3^/well) were cultured in 96-well plates and treated with VD extracts (10 and 50 μg/mL). The optical density at 490 nm was measured three times using a microplate reader (Sensident Scan).

### 2.5. Study of Animals and Their Diets

All animal experiment procedures were enforced in conformity to guidelines and with the approval of the Institutional Animal Care and Use Committees (IACUC) of Hallym University (Hallym-2015-12-R). Male C57BL/6N mice (five-week-old) were purchased from Central Lab Animal (SLC, Osaka, Japan). After one week’s rest, mice were discretionally allocated to one of four diet groups (six mice per group): normal fat diet (NFD), high-fat diet (HFD), HFD supplemented with 1% (10 g/kg) *Garcina cambogia* extract (GRD; ESFood, Gunpo, Korea), and HFD supplemented with 1% (10 g/kg) VDAE (VDD). *Garcinia cambogia* extract containing 60% (-)-hydroxycitric acid was used as a positive control because of its anti-adipogenic and anti-lipogenesis activities [17,18,19]. The experimental diets were based on the AIN-93 diet, and the HFD contained 60% fat (lard 310 g/kg, soybean oil 30 g/kg). *Garcina cambogia* extract and VDAE were dissolved in corn oil and added to the experimental diet. The diets of two groups were prepared by DooYeol Biotech (Seoul, Korea) and its compositions are shown in Table 1. Mice were housed under controlled temperature and lighting (22 ± 2 °C and 50 ± 10% humidity with a 12-h light/dark cycle) with free access to water and food. Mice followed the experimental diet for 10 weeks. Body weight was measured twice per week, and food intake was recorded every day.

### 2.6. Collection of Serum and Tissue Samples

After 10 weeks, all mice were sacrificed following 12-h fasting, and tissues were collected for analysis. Blood was collected from the inferior vena cava and separated immediately by centrifugation at 3000 rpm at 4 °C for 15 min to isolate the serum. The epididymal adipose tissue and liver were removed, weighed, and stored at 80 °C until analysis.

### 2.7. Biochemical Analysis

Levels of triacylglycerol (TG), high-density lipoprotein (HDL) cholesterol, low-density lipoprotein (LDL) cholesterol, serum alanine aminotransferase (ALT), aspartate aminotransferase (AST), blood urea nitrogen (BUN), and creatinine (CREA) in serum were measured with commercial kits (981786, 981823, 981656, 981769, 981771, 981820, and 981811, respectively, Thermo Electron Corporation, Vantaa, Finland) and a Thermo Fisher Konelab 20XTi Analyzer (Thermo Electron Corporation, SeoKwang LABOTECH, Seoul, Korea).

### 2.8. Histological Analysis

Epididymal adipose tissues were fixed with 4% formaldehyde and embedded in paraffin. Sections (5 μm thick) were cut and each section was stained with hematoxylin and eosin (H and E). All the sections were photographed using an optical microscope (Leica RM2235, Wetzlar, Germany) and printed at a final magnification of 200×. Images were observed with a microscope (Axiomager, Zeiss, Germany) and the diameter of each adipocyte was analyzed using AxioVisionRel. 4.8 software (Carl Zeiss, Oberkochen, Germany).

### 2.9. RNA Extraction, cDNA Synthesis and Real-Time PCR

Total RNA was extracted from the epididymal adipose tissue using an Easy-Blue kit (Intron Biotechnology Inc., Seoul, Korea) according to the protocol provided by the manufacturer. Then, total RNA was quantified with a NanoDrop-2000 (Thermo Fisher Scientific, Wilmington, DE, USA). cDNA was synthesized (0.03 μg of total RNA) with the Moloney murine leukemia virus transcriptase and Oligo (dT) 15 primers (Promega, Medison, WI, USA) using a Life Touch thermal cycler (Life Eco, Bioer Technology, Hangzhou, China). The program was set for 1 h of initiation at 42 °C, followed by 10 min of incubation at 95 °C and 10 min at 4 °C. RT-PCR was performed using the QuantiTect SYBR Green PCR kit (Qiagen), according to the manufacturer’s instructions. The cDNA (20 μL) was amplified for 40 cycles of denaturation (95 °C for 30 s), annealing (57 °C for 40 s), and extension (72 °C for 40 s) using a RotorGene RG3000 real-time PCR machine (Corbett Research, Sydney, Australia). The purity of the PCR products was determined using melting curve analysis. The relative quantification of the expression of each gene was calculated using the comparative threshold cycle (Ct) method (Applied Biosystems, Foster City, CA, USA). mRNA levels were normalized to β-actin. Primer sequences are shown in Table 2.

### 2.10. NMR-Based Hepatic Metabolomics

The NMR-based hepatic metabolomics including liver tissue preparation, pulse acquisition and metabolite identification, and data processing were performed according to previous reports with minor modifications [20,21]. The lipophilic extracts, which contained the lipid constituents of the liver, were used for ^1^HNMR spectroscopy. The liver tissue (0.1 g) was homogenized first in 1 mL chloroform/methanol (CHCl_3_/MeOH, 3:1, *v*/*v*). Then, after centrifugation at 10,000 rpm for 10 min at 4 °C, the supernatant was collected and dried under a stream of nitrogen. The lipophilic extracts were reconstituted with 665 μL of deuterated chloroform/methanol (CDCl_3_/CD_3_OD, 3:1, *v*/*v*) including tetramethylsilane (TMS) as an internal standard in NMR analysis. ^1^H NMR spectra of the isolated pure compounds were recorded using a Bruker AV 400 instrument.

### 2.11. Statistical Analysis

Data from individual experiments are expressed as a mean value ± SE and comparisons of data were carried out using a Student’s unpaired *t*-test or one-way ANOVA, as appropriate. *p* < 0.05 is considered statistically significant.

## 3. Results

### 3.1. Effect of VD Extracts on Cell Viability in Pre-Adipocytes

**Figure 1:**
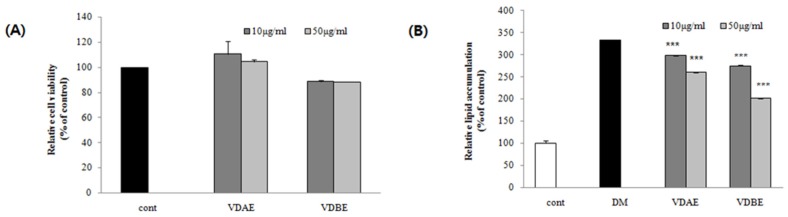
Effects of *Valeriana dageletiana* Nakai ex F. Maek. extracts in 3T3-L1. (**A**) Effect of VDAE and VDBE on the viability of 3T3-L1 cells determined by MTS assays at 10 and 50 μg/mL for 24 h; and (**B**) the relative lipid content quantified by absorbance in 3T3-L1 cells treatment with or without VDAE and VDBE at 10 and 50 μg/mL for eight days. Results are presented as means ± SE. The asterisk indicates a significant difference compared to DM (*** *p* < 0.001). Cont; Control, DM; Differentiation media cells, VDAE; extract from above-ground part of VD, VDBE; extract from the below-ground part of VD, VD; *Valeriana dageletiana* Nakai ex F. Maek.

An MTS assay was performed to determine the cell viability of VD extracts at concentrations of 10 and 50 μg/mL on 3T3-L1 cells. As shown in Figure 1A, the VDAE had no significant effects on viability after 24 h treatment; however, VDBE decreases cell viability by about 10%, indicating that VDBE would be cytotoxic to 3T3-L1 cells.

### 3.2. Inhibitory Effects of VD Extracts on Lipid Accumulation in 3T3-L1 Cells

The anti-adipogenic effects of VD extracts from different parts were compared using Oil Red O staining in differentiated 3T3-L1 cells at the concentration of 10 and 50 μg/mL. As shown in Figure 1B, treatment with VDAE and VABE significantly diminished in relative lipid contents in differentiated 3T3-L1 adipocytes. Among them, VDAE and ADBE exhibited the highest inhibitory effects (30.68% and 56.35%, respectively) on adipogenesis at 50 μg/mL.

### 3.3. Changes in Body Weight, Food Intake, and Food Efficiency Ratio (FER)

**Figure 2:**
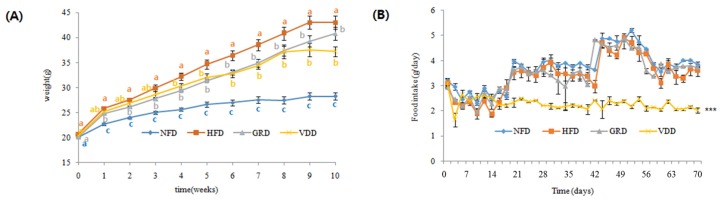
Effects of *Valeriana dageletiana* Nakai ex F. Maek. extract from the above-ground part (VDAE) on body weight (**A**) and food intake (**B**) in high-fat diet-induced obese mice. Results are presented as means ± SE (*n* = 6). NFD; normal fat diet control, HFD; high fat diet control, GRD; HFD + 1% *Garcina cambogia* extract, VDD; HFD + 1% *Valeriana dageletiana* Nakai ex F. Maek extract from above-ground part.

VDAE exhibited an anti-adipogenic effect on 3T3-L1 cells without cytotoxicity. Thus, VDAE was used for further anti-obesity studies in vivo. The compositions of experimental diets are listed in Table 1. There were no significant differences in body weight initially (Figure 2A); however, after 10 weeks of experimental diet feeding, the body weights of HFD mice increased by 2.90-fold than those of NFD mice. VDAE supplementation for 10 weeks significantly decreased body weights by 1.99-fold in comparison to the HFD group. In terms of food intake (Figure 2B), there was no significant difference initially; however, after three weeks, until to the end, food intake appeared to be influenced by VDAE supplementation. Therefore, as shown in Table 3, body weight gain was strongly suppressed by the administration of VDAE associated with significant decreases in food intake. Thus, compared to the other groups, the food efficiency ratio (FER) of the VDD group showed only a 1.34-fold decrease compared to those of HFD mice during the 10-week feeding period.

### 3.4. Measurement of Lipid Profiles and Potential Toxicity in Serum

The effects of HFD and VDAE administration on serum lipid profiles are shown in Table 4. The TG and total-cholesterol serum levels in the HFD group showed significant increases compared to that of the NFD group. However, there was a significant decrease in TG (2.16-fold) and total cholesterol levels (inhibition (%): 98.37) in the VDD group compared to those of the HFD group. Although HDL-cholesterol levels were reduced in the VDD group compared to in the HFD group, HTR was not significantly affected. The hepatic and renal toxicity of VDAE administration was confirmed by measuring serum AST, ALT, BUN, and CREA levels. AST, ALT, BUN, and CREA serum levels increased in the HFD group, and VDAE administration decreased AST, ALT, BUN, and CREA levels in comparison to the HFD group (inhibition (%): 57.40, 75.30, 56.26 and 53.33, respectively).

### 3.5. Changes in Weight and Morphology of Adipose Tissue

**Figure 3:**
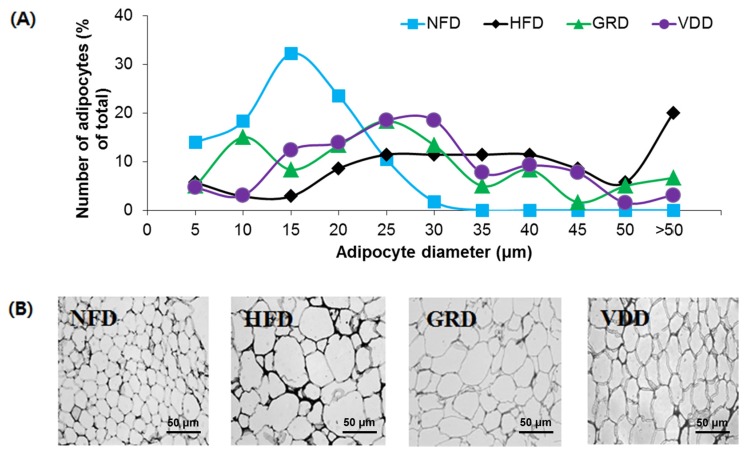
Effects of *Valeriana dageletiana* Nakai ex F. Maek extract from the above-ground part (VDAE) on epididymal white adipose tissue diameter (**A**) and microphology (**B**) in high-fat-fed mice. Each specimen from the mice groups was fixed with 4% paraformaldehyde and sectioned to 4 μm, stained with hematoxylin and eosin (H and E), and then viewed using light microscopy magnification, 200×. The image is a representative one. NFD; normal fat diet control, HFD; high fat diet control, GRD; HFD + 1% *Garcina cambogia* extract, VDD; HFD + 1% *Valeriana dageletiana* Nakai ex F. Maek extract from above-ground part.

To investigate whether the weight-reducing effect of VDAE was due to a decrease in fat mass, the adipocyte size and tissue weight were measured. The weight of epididymal white adipose tissue in the HFD group increased significantly compared to that in the NFD group (Table 3), and the adipocyte size was larger in the HFD group than in the NFD group (Figure 3). The weight of adipose tissue and the size of the adipocytes were clearly decreased by supplementation with VDAE in comparison to the HFD group (Table 3 and Figure 3).

### 3.6. Effect of VOD on the Expression of Genes Related to Adipogenesis and Lipogenesis in White Adipose Tissue

**Figure 4:**
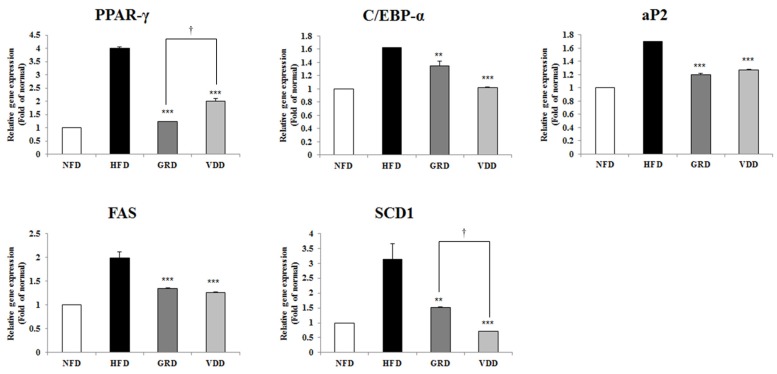
Effects of *Valeriana dageletiana* Nakai ex F. Maek extract from above-ground part (VDAE) on mRNA expression of adipogenic genes in epididymal white adipose tissue. Results are presented as means ± SE. The asterisk indicates a significant difference compared to HFD group (** *p* < 0.01, *** *p* < 0.001). The dagger indicates significant differences between GRD and VDD groups († *p* < 0.05). NFD; normal fat diet control, HFD; high fat diet control, GRD; HFD + 1% *Garcina cambogia* extract, VDD; HFD + 1% *Valeriana dageletiana* Nakai ex F. Maek extract from above-ground part.

To further evaluate VDAE mediated reduction in adipocyte size, we measured the expression of mRNA related to adipogenesis and lipogenesis. As shown in Figure 4, supplementation with VDAE significantly downregulated the expression of *PPAR-γ*, *C/EBP-α*, and *fatty acid binding protein 4 (aP2)*, which are all involved in adipogenesis. The expression of lipogenic genes, such as *FAS* and *SCD-1*, were also reduced by VDAE.

### 3.7. Changes in Liver Weight and Hepatic Lipid Metabolites

Since obesity is often accompanied with the development of fatty liver, we examined the effect of VDD on liver weight changes and hepatic lipid metabolites in HFD-fed mice. The liver weight in the HFD group was higher than in the NFD group, and VDAE supplementation significantly decreased liver weight by 1.51-fold (Table 3). Four types of hepatic lipid metabolites, including fatty acids, phospholipid, lipid moieties, and cholesterol, were analyzed, and their levels increased significantly in the HFD group (Table 5). Supplementation with VDAE significantly decreased the level of hepatic lipid metabolites. These results suggest that VDAE effectively inhibited lipid accumulation in the hepatic tissue.

### 3.8. Effect of VOD on the Expression of Genes Related to Lipogenesis in the Liver

**Figure 5:**
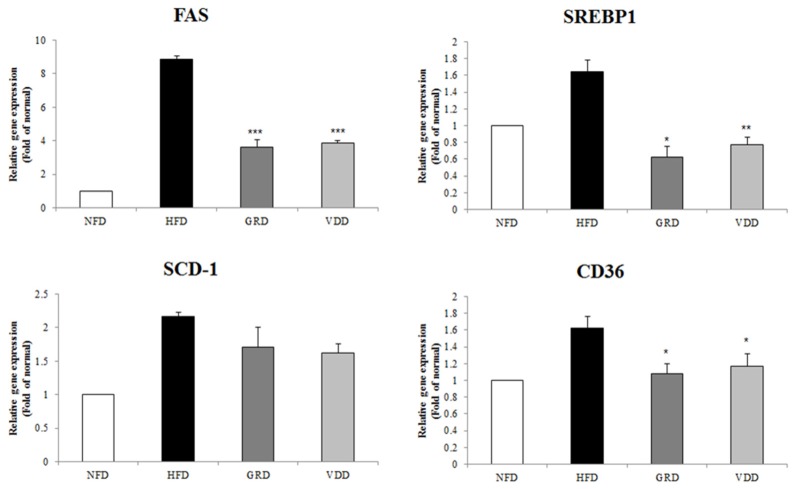
Effects of *Valeriana dageletiana* Nakai ex F. Maek extract from above-ground part (VDAE) on mRNA expression of lipogenesis-related genes in liver. Results are presented mean ± SE. The asterisk indicates a significant difference compared to HFD group (* *p* < 0.05, ** *p* < 0.01, *** *p* < 0.001). NFD; normal fat diet control, HFD; high fat diet control, GRD; HFD + 1% *Garcina cambogia* extract, VDD; HFD + 1% *Valeriana dageletiana* Nakai ex F. Maek extract from above-ground part.

To examine the effect of a decline in hepatic lipid accumulation with VDAE supplementation, we measured the expression of mRNA related to lipogenesis. VDAE supplementation significantly decreased the expression of lipogenesis-related genes, such as *SREBP-1c*, *FAS*, *SCD-1*, and *fatty acid translocase (CD36)*, compared with HFD in the hepatic tissue (Figure 5).

## 4. Discussion

In modern society, the number of people with obesity and obesity-related diseases, including diabetes mellitus, hypertension, diabetes, hyperlipidemia, non-alcoholic fatty liver diseases, and cancers, has increased worldwide [22,23,24]. Consequently, developing anti-obesity drugs with minimal side effects has become a popular theme [25]. Obesity is associated with the differentiation of pre-adipocytes, which is induced by adipogenesis, and accumulation of triglycerides in adipocytes. Thus, to prevent obesity, dietary supplements are used for the regulation of adipogenesis and lipogenesis. In this study, to corroborate the potential of VD extract as a food supplement for preventing obesity effect, we focused on regulation of adipogenesis and lipogenesis in vitro and in vivo.

The extraction of different parts of the plant can reveal specific bioactive ingredients and activities. Therefore, the extracts from above- and below-ground parts of VD were used to evaluate the anti-adipogenic activity in 3T3-L1 cells, and both VDAE and VDBE showed anti-adipogenic effects at a concentration of either 50 or 10 μg/mL. VDBE showed the strongest anti-adipogenic effect at a concentration of 50 μg/mL. It was also apparent that VDBE had a stronger anti-adipogenic effect than VDAE. However, VDBE was found to decrease the cell viability in the MTS assay, indicating that VDBE is potentially of cytotoxic to 3T3-L1 cells, whereas VDAE showed no significant cytotoxic effect on the cells. Moreover, the above part of VD is well known to be an edible part of the plant and, thus, VDAE was used for further anti-obesity studies in experimental obese mice.

Reducing body weight and fat are important in preventing obesity. Supplementation of HFD increased body weight gain along with cholesterol, TG, AST, ALT, and reduction of HTR in serum. VDAE clearly decreased food intake, body weight gain, and FER levels compared to the HFD group. Administration of VDAE also significantly decreased serum cholesterol, TG, AST, ALT, BUN, and CREA levels. It was suggested that the body weight-reducing effect of VDAE affects appetite. The decreased food intake may explain the anti-obesity effects of VDAE. Moreover, all the phenotypes, including lower body weight, better lipid profile, etc., can be considered the consequences of the lower food intake. Over the past decades, it is recognized that the food intake is not only regulated by the food flavor (i.e., palatable flavor of food can promote food intake), but it is also controlled by vagal and hormonal gut-brain signaling mechanisms (i.e., promote satiety and limit food intake) [26]. Some nutrients or special flavor foods are known simulating the satiety signals through the vagal and hormonal gut-brain signaling mechanisms involved in food intake regulation [27,28]. Resveratrol, the bioactive compound in grapes and red wine, was reported may represent promising novel treatment for obesity through affecting gut-brain signaling [29,30]. Epigallocatechin gallate, chitosan, hesperetin, and naringenin have also been found to have a similar effect as resveratrol on gut-brain signaling [31]. The food intake affected by VDAE may due to its special flavor regulating appetite through affecting the vagal and hormonal gut-brain signaling mechanisms. The reduction of body weight gain and serum lipids induced by VDAE administration can also be associated with the inhibition of fat accumulation without hepatic and renal toxicity.

Obesity is characterized by a separate or simultaneous increase in fat cell numbers and size. VDAE significantly decreased the epidermal white adipose tissue mass and the adipocyte size. Adipose tissue regulates fat cell development by coordinated binding of the transcription factors in the regulatory regions of adipogenesis-related genes. Several reports have indicated *PPAR-γ*, *C/EBP-α*, and *aP2* are the major transcriptional genes related to adipogenesis and lipogenesis genes, such as *FAS* and *SCD-1*. PPAR-γ is considered the major regulator of adipocyte differentiation and, along with *C/EBP-α*, it regulates the expression of downstream target genes [13,19,32]. We performed RT-PCR to analyze the molecular metabolism of VDAE-mediated adipogenesis and lipogenesis. HFD-induced expression of adipogenic genes such as *PPAR-γ* and *C/EBP-α*, which operate in a self-regulating positive feedback loop system and then increase the expression of adipogenic genes such as aP2 and lipogenic genes, such as *FAS* and *SCD-1*. aP2 is a carrier protein for fatty acids mainly expressed in adipocytes and macrophages and plays an important role in the development of insulin resistance and atherosclerosis in relation to metaflammation. aP2 is elevated by obesity and is used as a marker for adipocyte differentiation [33]. FAS and SCD-1 are the key enzymes involved in fatty acid metabolism responsible for synthesizing palmitate (C16:0) from acetyl-CoA and forming a double bond in stearoyl-CoA. However, VDAE administration reduced the expressions of these genes, including *PPAR-γ*, *C/EBP-α*, *aP2*, *FAS*, and *SCD-1*. These results suggest that VDD administration suppressed adipogenesis and lipogenesis by decreased transcription factors in adipose tissue.

**Figure 6:**
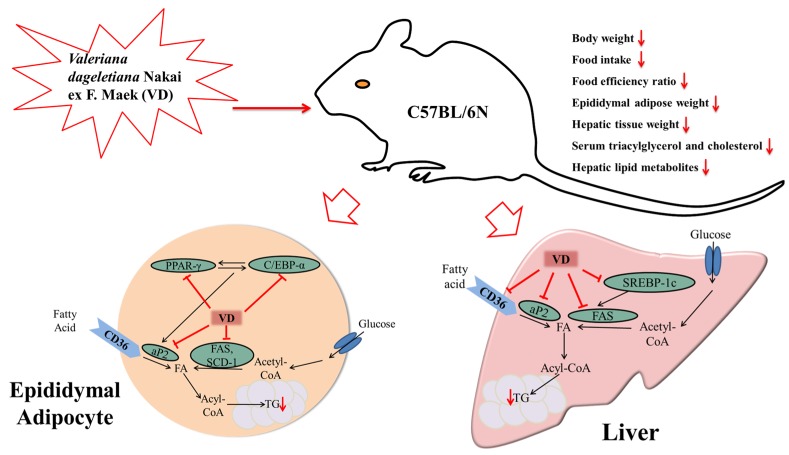
Proposed mechanisms of the anti-obesity effect of VDAE.

Since mobilization of hepatic lipids through lipogenesis causes an increase in fat mass and body weight gain, hepatic tissue is another important target for the prevention of obesity. The weight of hepatic tissue was markedly decreased by VDAE in comparison to the HFD group. In addition, the present study showed that lipogenesis-related genes (*SREBP-1c, FAS, SCD-1*) and hepatic lipid metabolites (*fatty acids, phospholipids, lipid moieties*) were clearly suppressed by VDAE administration in hepatic tissue. SREBP1c, a key player in hepatic lipogenesis, activates nearly all genes required for de novo synthesis of fatty acid and triglyceride synthesis [34], SCD-1 is the rate-limiting enzyme involved in the biosynthesis of monounsaturated fatty acids [35]. FAS is a central lipogenic protein that, along with CD36, contributes to energy storage by increasing fatty acid uptake in adipocytes and liver [36]. Thus, as shown in Figure 6, administration of VDAE suppressed synthesis of fatty acids, monounsaturated fatty acids, and triglyceride and fatty acid uptake in adipocytes and in the liver. Obesity, as a multifactorial and chronic disease, increases the risk of non-alcoholic fatty liver disease and liver injuries. Lipocalin-2 is an adipokine which plays roles in glucose metabolism, as well as inflammation. It was demonstrated that the liver is the main source of serum lipocalin-2 [37]. Previous reports suggested that lipocalin-2 may be a reliable biomarker of hepatic injury, inflammation and/or metabolic insult [38,39]. Thus, elevated lipocalin-2 levels can be observed in high-fat diet fed obese mice; and it will be interesting to examine the systemic levels of lipocaline-2 after administration of VDAE in further study to reveal the potential of VDAE on ameliorating liver injuries in high-fat diet-inducted obese mice. These results indicate that VDAE supplementation can contribute to preventing hepatic lipid accumulation through regulation of lipogenesis.

Generally, obesity and overweight could be prevented by physical activities and diet regulation (i.e., Mediterranean diet, avoiding or ameliorating the consumption of fructose, high-fat diet, and alcohol), which are the most natural ways and, thus, are normally recommended [40,41,42,43,44,45]. However, it is difficult to reverse the dietary and lifestyle trends of obese people in modern society. Nevertheless, they are too moderate to serious obesity patients. Bariatric surgery is considered as the effective procedure in improving insulin resistance and glucose metabolism for seriously obese patients, primarily by reducing calorie intake, consequently reducing body mass and liver steatosis [46,47]. Another idea is treatment with anti-obesity agents, such as metformin [48]. Alternatively, using herbal medicines or some plant foods, such as VDAE, may be a novel therapeutic approach for preventing or treating obesity.

## 5. Conclusions

In conclusion, we found that VDAE supplementation significantly suppressed lipid accumulation in 3T3-L1 adipocytes, as well as body weight gain, food intake, FER levels, weight, number, and size of adipose tissue, hepatic lipid metabolites, and serum levels of TG and cholesterol in C57BL/6N fed a high-fat diet. These results can be associated with the decrease in adipogenesis related to mRNA expression of *PPAR-γ*, *C/EBP-α*, and *aP2.* In addition, VDAE supplementation lowered the expression of lipogenic genes, such as *SREBP-1c, FAS, SCD-1,* and *CD36*. Hence, VDAE exerts anti-obesity effects by suppressing adipogenesis and lipogenesis and can be considered a potent and useful functional food resource to prevent obesity.

## Figures and Tables

**Table 1:** Compositions of experimental diets (g/kg).

Groups ^1^	NFD	HFD	GRD	VDD
Casein	210	265	265	265
L-cystine	3	4	4	4
Corn starch	280	-	-	-
Maltodextrin	50	160	150	150
Sucrose	325	90	90	90
Lard	20	310	310	310
Soybean Oil	20	30	30	30
Cellulose	37.15	65.5	65.5	65.5
Mineral Mixure ^2^	35	48	48	48
Vitamin Mixture ^3^	15	21	21	21
Calcium Phosphate, Diabasic	2	3.4	3.4	3.4
Choline Bitartrate	2.75	3	3	3
Yellow Food Color	0.1	-	-	-
Blue Food Color	-	0.1	0.1	0.1
*Garcinia cambogia* extract of 60% (-)-hydroxycitric acid	-	-	10	-
*Valeriana dageletiana* Nakai ex F. Maek extract from above-ground part	-	-	-	10
Total (g)	1000	1000	1000	1000

^1^ NFD; normal fat diet control, HFD; high fat diet control, GRD; HFD + 1% *Garcinia cambogia* extract, VDD; HFD + 1% *Valeriana dageletiana* Nakai ex F. Maek extract from above-ground part. ^2^ Mineral mixture is according to AIN-93G-MX (94046). ^3^ Vitamin mixture is according to AIN-93-VX (94047).

**Table 2:** Sequence of primers used in real-time PCR.

Gene	Primer Sequence (5’→3’)	
	Forward Primer	Reverse Primer
*β-Actin*	GTCGTACCACTGGCATTGTG	GCCATCTCCTGCTCAAAGTC
*C/EBP-α*	AGACATCAAGCGCCTACATCG	TGTAGGTGCATGGTGGTCTG
*PPAR-γ*	CCCTGGCAAACGATTTGTAT	AATCCTTGGCCCTCTGAGAT
*SREBP-1c*	GCGCTACCGGTCTTCTATCA	TGCTGCCAAAAGACAAGGG
*CD36*	TCCTCTGACATTTGCAGGTCTATC	GTGAATCCAGTTATGGGTTCCAC
*SCD-1*	CGAGGGTTGGTTGTTGATCTGT	ATAGCACTGTTGGCCCTGGA
*FAS*	GATCCTGGAACGAGAACAC	AGACTGTGGAACACGGTGGT
*aP2*	AACACCGAGATTTCCTTCAA	TCACGCCTTTCATAACACAT

**Table 3:** Effects of *Valeriana dageletiana* Nakai ex F. Maek extract from the above-ground part (VDAE) on body weight in mice fed a high-fat diet.

Groups ^1^	NFD	HFD	GRD	VDD
Body weight gain (g/10 weeks)	8.29 ± 0.74 ^a,2^	24.05 ± 1.28 ^b^	19.27 ± 1.28 ^c^	12.06 ± 0.81 ^d^
Food intake (g/day)	3.74 ± 0.13 ^a^	3.41 ± 0.13 ^a^	3.51 ± 0.13 ^a^	2.28 ± 0.04 ^b^
FER ^3^	2.22 ± 0.2 ^a^	7.06 ± 5.49 ^b^	5.49 ± 0.36 ^c^	5.27 ± 0.36 ^c^
Epididymal white adipose tissue weight (g)	1.00 ± 0.16 ^a^	2.31 ± 0.16 ^b^	2.18 ± 0.16 ^b^	2.09 ± 0.09 ^b^
Liver weight (g)	1.27 ± 0.19 ^a^	1.65 ± 0.38 ^a^	1.19 ± 0.07 ^a^	1.09 ± 0.07 ^a^

^1^ NFD; normal fat diet control, HFD; high fat diet control, GRD; HFD + 1% *Garcinia cambogia* extract, VDD; HFD + 1% *Valeriana dageletiana* Nakai ex F. Maek extract from the above-ground part. ^2^ Results are presented as means ± SE (*n* = 6); values within arrow with different letters are significantly different from each other at *p* < 0.05. ^3^ Food efficiency ratio (FER) = Body weight gain (g/day)/Food intake (g/day).

**Table 4:** Effects of *Valeriana dageletiana* Nakai ex F. Maek extract from the above-ground part (VDAE) on obese biomarkers in mice fed a high-fat diet.

Groups ^1^	NFD	HFD	GRD	VDD
Serum TG (mg/dL)	115.5 ± 11.6 ^a,2^	120.9 ± 13.7 ^a^	120.3 ± 8.4 ^a^	55.92 ± 4.92 ^b^
Serum total-cholesterol (mg/dL)	53.52 ± 4.23 ^b^	118.1 ± 5.67 ^a^	127.12 ± 8.30 ^a^	54.57 ± 2.8 ^b^
Serum HDL-cholesterol (mg/dL)	43.17 ± 2.79 ^b^	78.81 ± 3.16 ^a^	79.45 ± 2.38 ^a^	38.63 ± 1.46 ^b^
HTR ^3^	0.81 ± 0.02 ^a^	0.70 ± 0.01 ^c^	0.66 ± 0.02 ^b^	0.71 ± 0.01 ^c^
AST (U/L)	66.54 ± 15.29 ^b,c^	79.61 ± 15.78 ^a,b^	113.69 ± 15.75 ^a^	33.93 ± 2.3 ^c^
ALT (U/L)	31.85 ± 7.75 ^b^	89.52 ± 22.90 ^a^	70.43 ± 11.76 ^a,b^	22.11 ± 5.57 ^b^
BUN (mg/dL)	22.55 ± 0.78 ^a^	17.49 ± 0.32 ^b^	17.73 ± 0.47 ^b^	7.65 ± 0.6 ^c^
CREA (mg/dL)	0.41 ± 0.02 ^b^	0.45 ± 0.01 ^a^	0.42 ± 0.01 ^b^	0.21 ± 0.01 ^c^

^1^ NFD; normal fat diet control, HFD; high fat diet control, GRD; HFD + 1% *Garcinia cambogia* extract, VDD; HFD + 1% *Valeriana dageletiana* Nakai ex F. Maek extract from above-ground part. ^2^ Results are presented as means ± SE (*n* = 6); values within arrow with different letters are significantly different from each other at *p* < 0.05. ^3^ HTR = HDL-cholesterol/total-cholesterol.

**Table 5:** ^1^H NMR chemical shifts for endogenous lipid-soluble hepatic metabolites.

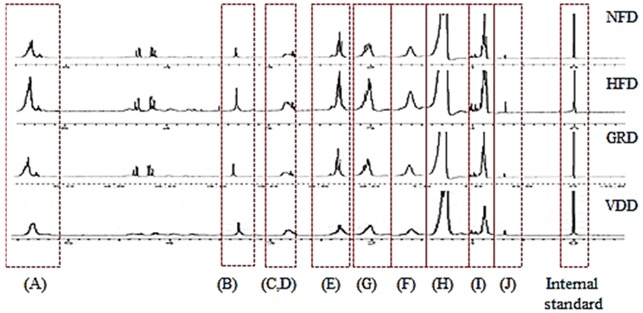								
	δ ^1^H (ppm)	Type	Metabolites	Group ^1^				
				NFD		HFD	GRD	VDD
(A)	5.32	Fatty acids	MUFA/PUFA		7.78 ± 0.80 ^a^	29.8 ± 4.38 ^b^	9.63 ± 1.76 ^a^	6.71 ± 0.90 ^a^
(B)	3.16	Phospholipid	N^+^(CH_3_)_3_		0.18 ± 0.00 ^a^	2.11 ± 0.34 ^b^	0.33 ± 0.00 ^c^	0.23 ± 0.01 ^d^
(C)	2.78	Lipid moieties	-CH=CH(-CH_2_CH=CH-)	}	3.56 ± 0.29 ^a^	18.2 ± 4.98 ^b^	3.96 ± 0.14 ^a^	3.2 ± 0.37 ^a^
(D)	2.72	Lipid moieties	-CH=CH-CH_2_CH=CH-					
(E)	2.27	Lipid moieties	αRCH_2_CH_2_CO		7.4 ± 1.02 ^a^	31.67 ± 9.92 ^b^	10.61 ± 2.46 ^a,b^	6.17 ± 0.10 ^a^
(F)	2.00	Lipid moieties	-CH_2_CH=CH-		6.92 ± 0.43 ^a^	39.22 ± 11.61 ^b^	12.91 ± 3.26 ^a,b^	9.1 ± 1.02 ^a^
(G)	1.55	Lipid moieties	βRCH_2_CH_2_CO		6.49 ± 0.20 ^a^	23.61 ± 2.53 ^b^	10.17 ± 2.48 ^a^	6.59 ± 1.06 ^a^
(H)	1.24	Lipid moieties	-(CH_2_)_n_-		54.65 ± 3.47 ^a^	201.94 ± 32.30 ^b^	78.24 ± 21.31 ^a^	48.02 ± 9.28 ^a^
(I)	0.83	Lipid moieties	RCH_3_		4.6 ± 0.55 ^a^	33.57 ± 7.76 ^c^	10.63 ± 2.27 ^b^	7.53 ± 1.43 ^a,b^
(J)	0.64	Cholesterol	Chol-C18		0.05 ± 0.03 ^a^	0.51 ± 0.35 ^a^	0.20 ± 0.73 ^a^	0.17 ± 0.39 ^a^

^1^ NFD; normal fat diet control, HFD; high fat diet control, GRD; HFD + 1% *Garcinia cambogia* extract, VDD; HFD + 1% *Valeriana dageletiana* Nakai ex F. Maek extract from above-ground part. Results are presented as means ± SE (*n* = 6); values within arrow with different letters are significantly different from each other at *p* < 0.05.
